# Mentorship in General Internal Medicine: Results from a National Survey

**DOI:** 10.1007/s11606-025-09862-3

**Published:** 2025-10-02

**Authors:** Vineet Chopra, M. Todd Greene, Jason M. Engle, Karen E. Fowler, Sanjay Saint

**Affiliations:** 1 The Department of Medicine, University of Colorado Anschutz Medical Campus, Aurora, CO USA; 2 The VA Ann Arbor Center for Clinical Management Research, Ann Arbor, MI USA; 3 The Department of Internal Medicine, University of Michigan, Ann Arbor, MI USA

## INTRODUCTION

Mentorship in the workplace involves a senior individual guiding a junior colleague. Although mentorship is linked to career progression, higher morale, and reduced turnover,^1^ its prevalence and influence in general internal medicine (GIM) are unclear. Physicians with mentors may better navigate work structures and experience lower burnout. Mentorship could also enhance autonomy, mastery, and purpose, motivating individuals in their work.^2,3^ However, factors influencing who finds a mentor and the impacts of such mentorship on outcomes are poorly understood. We sought to define the prevalence and effect of mentorship among GIM physicians.

## METHODS

A nationwide, cross-sectional survey of GIM physicians was conducted between June 2023 and May 2024 using the American Medical Association Physician Professional Data™. Our survey targeted physician well-being across clinical environments.^4^ In addition to questions on mentorship, the survey included the 22-item Maslach Burnout Inventory.^5^ The University of Michigan Institutional Review Board deemed this study exempt from regulation.

Descriptive statistics were used to describe respondent characteristics. Chi-square tests for categorical variables and t-tests for continuous variables were used to assess associations between respondent characteristics and mentorship. Informed by the theory of self-determination,^6^ we explored how mentorship influences elements such as autonomy, mastery, empowerment, purpose, and burnout. Burnout was defined, by convention, as meeting thresholds in all three domains of the Maslach Burnout Inventory.^7^ Multivariable regression models adjusting for race, sex, and years of practice were fit to examine associations between mentorship and these aspects. Results were reported as odds ratios (OR) with corresponding 95% confidence intervals; *p* < 0.05 was considered significant.

## RESULTS

Of 1421 invited physicians, 629 responded (response rate = 44.3%). Respondents were predominantly male (60.9%), white (59.2%) and practiced medicine for a median (IQR) of 23 (15–29) years (see Table 1). With respect to the setting of care, 44.8% reported working at a community medical center or clinic, 30.1% at a Veterans Affairs medical center, and 13.9% at an academic medical center or clinic. Hospitalists comprised 48.5% and primary care providers made up 49.7%.

**Table 1:** Characteristics of Internal Medicine Physician Study Participants (*N* = 629)

Characteristics	N (%)
Median time spent in practice, years (IQR)	23 (15–29)
Median time spent working per week, hrs (IQR)	50 (40–70)
Male	376/617 (60.9)
Race/ethnicity	
White	362/611 (59.2)
Asian	189/611 (30.9)
Black or African American	31/611 (5.1)
Other	26/611 (4.3)
Native Hawaiian or Pacific Islander	2/611 (0.3)
American Indian or Alaska Native	1/611 (0.2)
Marital status	
Married or living as if married	534/618 (86.4)
Single, never married	33/618 (5.3)
Divorced	33/618 (5.3)
Separated	11/618 (1.8)
Widowed	7/618 (1.1)
Children under age 18 years	266/618 (43.0)
Clinical work setting	
Inpatient setting only	254/623 (40.8)
Outpatient setting only	236/623 (37.9)
Both inpatient and outpatient	100/623 (16.1)
Other	33/623 (5.3)
Type of facility for majority of clinical work	
Community medical center or clinic	277/618 (44.8)
VA medical center or clinic	186/618 (30.1)
Academic medical center or clinic	86/618 (13.9)
Other	69/618 (11.2)
Role*	
Hospitalist	299/617 (48.5)
Provider of primary care	307/618 (49.7)
Have professional mentor(s)	168/622 (27.0)
Excellent support from mentor(s)	52/166 (31.3)
Extreme burnout^a^	61/622 (9.8)
Strong sense of purpose in life^b^	326/617 (52.8)
Strong sense of purpose in work^c^	313/615 (50.9)
Work-life balance^d^	84/626 (13.4)
Work mastery^e^	70/624 (11.2)
Work autonomy^f^	102/627 (16.3)

^*^These percentages do not equal 100%, because two separate dichotomous questions were posed: 1) Do you consider yourself a hospitalist? 2) Do you provide primary care? ^a ^Extreme Burnout definition = a dichotomous variable coded as 1 if respondent scored high in both the emotional exhaustion (≥ 27) and depersonalization (≥ 10) domains and low in the personal accomplishment (≤ 33) domain of the Maslach Burnout Inventory, coded 0 otherwise. ^b ^Strongly agree with the statement, “I have a strong sense of purpose in my life.” ^c ^Strongly agree with the statement, “I have a strong sense of purpose in my work.” ^d^ Strongly agree with the statement, “My work schedule leaves me enough time for my personal/family life.” ^e^ Strongly agree with the statement, “I feel as if someday I can master my work.” ^f^ Strongly agree with the statement, “I have great autonomy over my work.”

Among respondents, 27.0% had a mentor; 37.7% in academic settings versus 23.5% in community settings (*p* = 0.01). Prevalence did not significantly differ by sex (*p* = 0.84) or hospitalist status (*p *= 0.84). Mentored individuals had fewer years in practice (*p *= 0.002) and were more often white (*p* = 0.01). Of those mentored, 31.3% reported excellent support. Older and male respondents more often indicated strong mentorship support (*p* < 0.001 and *p* = 0.07, respectively).

After adjusting for race, sex, and years of practice, those with mentors had significantly increased odds of reporting a strong sense of purpose in life (OR = 2.81, *p* < 0.001) and in work (OR = 2.20, *p* < 0.001) vs. those without mentorship (see Table 2). Having a mentor was also significantly associated with increased odds of autonomy in work (OR = 1.92, *p* = 0.01). Statistically significant associations between having a mentor and work-life balance (OR = 1.43, *p* = 0.18) and mastery at work (OR = 1.50, *p* = 0.14) were not detected. Finally, those with mentors demonstrated reduced odds of burnout vs. those without (OR = 0.49, *p* = 0.049).

**Table 2:** Multivariable Associations Between Mentorship and Physician Well-Being Characteristics

Characteristic	OR	95% CI	P-value
***Primary predictor:*** ***Having a mentor***			
Extreme burnout^a^	0.49	0.24–1.00	0.049
Strong sense of purpose in life^b^	2.81	1.89–4.19	< 0.001
Strong sense of purpose in work^c^	2.20	1.49–3.25	< 0.001
Work-life balance^d^	1.43	0.85–2.41	0.18
Work mastery^e^	1.50	0.87–2.58	0.14
Work autonomy^f^	1.92	1.21–3.05	0.01
***Primary predictor:*** ***Having excellent support from mentor***			
Extreme burnout^a^	0.24	0.03–2.04	0.19
Strong sense of purpose in life^b^	2.44	1.03–5.77	0.04
Strong sense of purpose in work^c^	1.66	0.76–3.64	0.20
Work-life balance^d^	1.65	0.66–4.13	0.28
Work mastery^e^	1.57	0.61–4.04	0.35
Work autonomy^f^	3.90	1.71–8.87	0.001

All models adjusted for race, sex, and number of years practicing as an internist. ^a ^Extreme Burnout definition = a dichotomous variable coded as 1 if respondent scored high in both the emotional exhaustion (≥ 27) and depersonalization ( ≥ 10) domains and low in the personal accomplishment (≤ 33) domain of the Maslach Burnout Inventory, coded 0 otherwise. ^b ^Strongly agree with the statement, “I have a strong sense of purpose in my life.” ^c ^Strongly agree with the statement, “I have a strong sense of purpose in my work.” ^d^ Strongly agree with the statement, “My work schedule leaves me enough time for my personal/family life.” ^e ^Strongly agree with the statement, “I feel as if someday I can master my work.” ^f^ Strongly agree with the statement, “I have great autonomy over my work.”

Respondents who indicated receiving excellent support from mentors more often indicated having a strong sense of purpose in life (OR = 2.44, *p *= 0.04) and having greater work autonomy (OR = 3.90, *p* = 0.001). No significant associations between excellent support from mentors and strong agreement with purpose in work (OR = 1.66, *p* = 0.20), work-life balance (OR = 1.65, *p* = 0.28), work mastery (OR = 1.57, *p* = 0.35), or burnout (OR = 0.24, *p* = 0.19) were detected.

## DISCUSSION

This study is among the first to examine mentorship prevalence in GIM physicians across diverse settings. Despite previous calls to increase GIM mentoring, our findings suggest it remains uncommon. Yet when such relationships exist, physicians experience a stronger sense of autonomy, purpose, and well-being, with lower burnout risk. While our study cannot establish causality, promoting formal mentoring relationships in GIM is likely beneficial.

## Data Availability

Deidentified data will be made available upon reasonable request and ethical approval (e-mail: mtgreene@med.umich.edu).
